# Depression and anxiety in a case series of amyotrophic lateral sclerosis: frequency and association with clinical features

**DOI:** 10.1590/S1679-45082017AO3870

**Published:** 2017

**Authors:** Laura de Godoy Rousseff Prado, Isabella Carolina Santos Bicalho, Mauro Vidigal-Lopes, Vitor de Godoy Rousseff Prado, Rodrigo Santiago Gomez, Leonardo Cruz de Souza, Antônio Lúcio Teixeira

**Affiliations:** 1 Universidade Federal de Minas Gerais, Belo Horizonte, MG, Brazil.; 2 Fundação Hospitalar do Estado de Minas Gerais, Hospital Júlia Kubitschek, Belo Horizonte, MG, Brazil.

**Keywords:** Amyotrophic lateral sclerosis/diagnosis, Depression, Anxiety

## Abstract

**Objective:**

To investigate the frequency of anxiety and depression and their association with clinical features of amyotrophic lateral sclerosis.

**Methods:**

This is a cross-sectional and descriptive study including a consecutive series of patients with sporadic amyotrophic lateral sclerosis according to Awaji’s criteria. Patients underwent clinical and psychiatric assessment (anxiety and depression symptoms).

**Results:**

We included 76 patients. The men/women ratio was 1.6:1. Participants’ mean age at disease onset was 55 years (SD±12.1). Sixty-six patients (86.8%) were able to complete psychiatric evaluation. Clinically significant anxiety was found in 23 patients (34.8%) while clinically significant depression was found in 24 patients (36.4%). When we compared patients with and without depression a significant difference was seen only in the frequency of anxiety symptoms (p<0.001). We did further analysis comparing subgroups of patients classified according to the presence or not of anxiety and or depression, without any significant difference regarding sex, age at onset, initial form, disease duration or functional measures. A positive correlation between anxiety and depressive symptoms was found (p<0.001).

**Conclusion:**

Anxiety and depressive symptoms were highly correlated and frequent in patients with amyotrophic lateral sclerosis. In addition, anxiety and depression were not associated with disease duration and presentation, sex, age at onset, and functional score.

## RESUMO

**Objetivo:**

Investigar a frequência de ansiedade e depressão e sua associação com aspectos clínicos da esclerose lateral amiotrófica.

**Métodos:**

Estudo transversal e descritivo de uma série consecutiva de pacientes com esclerose lateral amiotrófica esporádica conforme os critérios de Awaji. Os pacientes foram submetidos à avaliação clínica e psiquiátrica (sintomas depressivos e ansiosos).

**Resultados:**

Foram incluídos 76 pacientes. A relação homem/mulher foi de 1,6:1. A média de idade de início dos sintomas foi de 55 anos (DP±12,1). Foram capazes de completar a avaliação psiquiátrica 66 (86,8%) pacientes. Ansiedade clinicamente significativa foi encontrada em 23 pacientes (34,8%), enquanto depressão clinicamente significativa foi encontrada em 24 pacientes (36,4%). Ao comparar os pacientes com e sem depressão, houve diferença significativa apenas na frequência de sintomas de ansiedade (p<0,001). Posteriormente, foram comparados subgrupos de pacientes categorizados em relação à presença ou não de ansiedade e/ou depressão, sem diferença significativa em relação a sexo, idade de início dos sintomas, forma inicial, duração da doença ou na escala funcional. Foi encontrada correlação positiva entre os sintomas de ansiedade e depressão (p<0,001).

**Conclusão:**

Sintomas de ansiedade e depressão são frequentes em pacientes com esclerose lateral amiotrófica e estiveram altamente correlacionados. Ansiedade e depressão não foram associadas com duração da doença, forma inicial, sexo, idade de início dos sintomas e pontuação na escala funcional.

## INTRODUCTION

Amyotrophic lateral sclerosis (ALS) is characterized by motor neuron degeneration leading to progressive muscle paresis and functional disability, with a mean survival of 3 to 5 years.^(1)^ Non-motor symptoms may be seen including depression and anxiety.^(1,2)^ The prevalence of depression and anxiety is highly variable among published studies, and may be present in up to 44 and 30% of patients, respectively.^(2)^ There is evidence indicating that functional disability and depression are linked, and that depression reduces the quality of life of patients with ALS.^(3)^ However, some studies failed to confirm the association between the severity of anxiety and depressive symptoms, and functional disability in ALS.^(4,5)^

This study investigated clinical features and anxiety/depressive symptoms in Brazilian patients with ALS. We hypothesized that the presence of anxiety and depression would be related to a more significant functional impairment.

## OBJECTIVE

To investigate the frequency of anxiety and depression and their association with clinical features of amyotrophic lateral sclerosis.

## METHODS

This is a cross-sectional study of consecutive patients diagnosed with probable or definite sporadic ALS according to Awaji’s criteria^(6)^ conducted from May 2013 to November 2015. All patients signed the written consent.

The protocol included standardized questions regarding clinical features of ALS, the Hospital Anxiety and Depression Scale (HADS)^(7,8)^ and the ALS Functional Rating Scale Revised (ALSFRS-R).^(9)^ A total score on HADS for depression above 8 is highly indicative of clinically significant depression (*i.e*. probable depression); the same criteria was applied for anxiety.^(8)^

The study was approved by the human research Ethics Committee of both *Universidade Federal de Minas Gerais*, CAAE: 19599813.0.0000.5149 and *Fundação Hospitalar do Estado de Minas Gerais,* protocol number 002B/2014.

### Statistical analysis

We performed non-parametric tests (Mann-Whitney, χ^2^ and Spearman) using Statistical Package for the Social Sciences (SPSS), version 22.0. A p value <0.05 was considered statistically significant.

## RESULTS

Seventy-six patients were selected for the study. The clinical parameters are summarized at table 1. Forty-seven patients (61.8%) were men (men/women ratio: 1.6:1). Considering the initial presentation, 78% of cases were spinal and 22% were bulbar.

**Table 1:** Descriptive analysis of clinical parameters of patients with amyotrophic lateral sclerosis

Sporadic ALS (n=76)	Mean (SD±)	Median	Minimun	Maximun
Age at the onset of symptoms, years	55.0 (12.1)	55	26	82
Age at assessment, years	58.3 (11.6)	58	32	83
Disease duration until assessment, years	3.32 (2.8)	2	0	14

ALS: amyotrophic lateral sclerosis; SD: standard deviation.

Sixty-six patients (86.8%) completed the HADS (Table 2). The others did not fulfill the scale due to refusal (n=4) or severe disability (n=6). Probable anxiety was confirmed in 23 (34.8%) patients; probable depression was found in 24 (36.4%) patients. Of 66 patients, 65% were men, 50 (75%) had spinal and 16 (24%) had bulbar initial presentation. Fifteen (23%) had anxiety and depression, 9 (14%) had only depression, 8 (12%) had only anxiety, while 34 (51%) had none of these symptoms. Thirty patients were current using antidepressants to treat depressive state and/or pain, insomnia or excessive salivation. Eleven of 24 patients with depression (45.8%) were using effective doses of antidepressants.

**Table 2:** Descriptive analysis of clinical parameters of patients with amyotrophic lateral sclerosis who completed Hospital Anxiety and Depression Scale

Sporadic ALS (n=66)	Mean (SD±)	Median	Minimun	Maximun
Age at the onset, years	54.3 (12.1)	55	26	82
Age at assessment, years	57.6 (11.5)	57,5	32	83
Score on ALSFRS-R	26.9 (10.1)	27	0	46
Score on HADS-anxiety	7.2 (4.4)	6	0	17
Score on HADS-depression	6.7 (4.5)	7	0	18

ALS: amyotrophic lateral sclerosis; ALSFRS-R: amyotrophic lateral sclerosis functional rating scale revised; HADS: hospital anxiety and depression scale; SD: standard deviation.

When we compared subgroups of patients according to the presence or not of probable depression, there was a statistical difference regarding the score on HADS for anxiety (Table 3). No significant difference was found regarding sex and initial form.

**Table 3:** Comparative analysis of subgroups of patients, according to presence or the absence of significant depressive symptoms (Hospital Anxiety and Depression Scale for depression ≥9)

	Depression				
	
	Yes		No		p value
	
	n	mean (r)	n	mean (r)	
Years of study	24	6.4 (3-19)	42	7.3 (0-20)	0.66
Age at the onset of disease, years	24	58.1 (41-76)	42	52.1 (26-82)	0.06
Anxiety	24	10.8 (4-17)	42	5.2 (0-15)	0.00*
Score on ALSFRS-R	24	25.8 (0-46)	42	27.6 (4-46)	0.44
Disease duration, years	24	3.0 (0-11)	42	3.4 (0-14)	0.60

*p<0.05.

n: number of patients; r: range; ALSFRS-R: amyotrophic lateral sclerosis functional rating scale revised.

We did further analysis comparing subgroups of patients categorized according to the presence or not of anxiety and or depression, and no significant difference regarding ALSFRS-R scores, sex, age at onset, disease duration and initial form emerged.

There was no correlation between HADS scores for anxiety or depression and ALSFRS-R scores. A positive correlation between anxiety and depressive symptoms was found (r=0.67; p<0.001).

## DISCUSSION

To the best of our knowledge, this is the first study assessing psychiatric symptoms in ALS patients from South America. The frequency of depression (zero to 44%) and anxiety (zero to 30%) in ALS varies significantly across the studies.^(2,3,4)^ Our rates (around 35%) are within the range reported in the literature.

The assessment of psychiatric symptoms in ALS may be hampered by several factors, including assessment tools^(10)^ and severity of the disease,^(3,11)^ partly explaining the discrepancy among studies. Moreover, the use of antidepressants, that is frequent in ALS for different reasons,^(2-4,12)^ may have influenced the frequency and/or severity of depressive and anxiety symptoms.

Interestingly, despite the use of antidepressants, psychiatric symptoms are prominent, indicating persistence of symptoms even with treatment.^(2,3,12)^ It remains to be defined whether these patients respond differently to antidepressants and which factors are associated to clinical response.

Contrary to our hypothesis, we did not find significant association between symptoms of anxiety/depression and functional disability. The absence of association between depression and disease duration or functional score in ALS were reported in some studies,^(4,5)^ but other authors did find this association.^(3)^ Methodological issues, including differences in the selection criteria of patients and in the choice of the functional and psychiatric scales may account for these distinct findings. The controversial findings in literature hamper a precise conclusion about this subject. Further studies could help to clarify this data.

Based on the absence of significant difference in clinical features between subgroups, it is possible that these psychiatric symptoms may be due to other reasons not related to motor and functional impairment. It also remains to be elucidated why anxiety and depression symptoms were highly correlated in ALS. Limitations of the study include sample size, bias of the use of antidepressants and intrinsic limitations of the HADS.

## CONCLUSION

The frequency of anxiety and depression in amyotrophic lateral sclerosis was elevated, and was not associated with disease duration, sex, presentation form and functional score. There was a high correlation between anxiety and depression symptoms in amyotrophic lateral sclerosis.
